# *TERT* promotor status does not add prognostic information in *IDH*-wildtype glioblastomas fulfilling other diagnostic WHO criteria: A report of the RANO *resect* group

**DOI:** 10.1093/noajnl/vdac158

**Published:** 2022-09-29

**Authors:** Philipp Karschnia, Jacob S Young, Antonio Dono, Levin Häni, Stephanie T Juenger, Tommaso Sciortino, Francesco Bruno, Nico Teske, Ramin A Morshed, Alexander F Haddad, Yalan Zhang, Sophia Stoecklein, Michael A Vogelbaum, Juergen Beck, Nitin Tandon, Shawn Hervey-Jumper, Annette M Molinaro, Roberta Rudà, Lorenzo Bello, Oliver Schnell, Yoshua Esquenazi, Maximilian I Ruge, Stefan J Grau, Martin van den Bent, Michael Weller, Mitchel S Berger, Susan M Chang, Joerg-Christian Tonn

**Affiliations:** Department of Neurosurgery, Ludwig-Maximilians-University, Munich, Germany; German Cancer Consortium (DKTK), Partner Site Munich, Munich, Germany; Department of Neurosurgery & Division of Neuro-Oncology, University of San Francisco, San Francisco, California, USA; Department of Neurosurgery, McGovern Medical School at UT Health Houston, Houston, Texas, USA; Department of Neurosurgery, University of Freiburg, Freiburg, Germany; Department of Neurosurgery, University of Cologne, Cologne, Germany; Division for Neuro-Oncology, Department of Oncology and Hemato-Oncology, University of Milan, Milan, Italy; Division of Neuro-Oncology, Department of Neuroscience, University of Turin, Turin, Italy; Department of Neurosurgery, Ludwig-Maximilians-University, Munich, Germany; German Cancer Consortium (DKTK), Partner Site Munich, Munich, Germany; Department of Neurosurgery, McGovern Medical School at UT Health Houston, Houston, Texas, USA; Department of Neurosurgery & Division of Neuro-Oncology, University of San Francisco, San Francisco, California, USA; Department of Neurosurgery & Division of Neuro-Oncology, University of San Francisco, San Francisco, California, USA; Department of Neurosurgery & Division of Neuro-Oncology, University of San Francisco, San Francisco, California, USA; Department of Radiology, University Hospital, LMU Munich, Munich, Germany; Department of NeuroOncology, Moffitt Cancer Center, Tampa, Florida, USA; Department of Neurosurgery, University of Freiburg, Freiburg, Germany; Department of Neurosurgery & Division of Neuro-Oncology, University of San Francisco, San Francisco, California, USA; Department of Neurosurgery & Division of Neuro-Oncology, University of San Francisco, San Francisco, California, USA; Division of Neuro-Oncology, Department of Neuroscience, University of Turin, Turin, Italy; Division of Neurology, Castelfranco Veneto and Treviso Hospital, Treviso, Italy; Division for Neuro-Oncology, Department of Oncology and Hemato-Oncology, University of Milan, Milan, Italy; Department of Neurosurgery, University of Freiburg, Freiburg, Germany; Department of Neurosurgery, McGovern Medical School at UT Health Houston, Houston, Texas, USA; Department of Stereotactic and Functional Neurosurgery, Centre for Neurosurgery, University Hospital Cologne, Cologne, Germany; Department of Neurosurgery, University of Cologne, Cologne, Germany; Klinikum Fulda, Academic Hospital of Marburg University, Fulda, Germany; Department of Neurology, Erasmus MC Cancer Institute, Rotterdam, The Netherlands; Department of Neurology, University Hospital and University of Zurich, Zurich, Switzerland; Department of Neurosurgery & Division of Neuro-Oncology, University of San Francisco, San Francisco, California, USA; Department of Neurosurgery & Division of Neuro-Oncology, University of San Francisco, San Francisco, California, USA; Department of Neurosurgery, Ludwig-Maximilians-University, Munich, Germany; German Cancer Consortium (DKTK), Partner Site Munich, Munich, Germany

In **IDH**-wildtype glioblastomas which meet the histopathological or molecular diagnosis criteria, it remains unclear whether the presence of **TERT** promotor mutations provides additional prognostic information. Based on a multicenter cohort of 466 **IDH**-wildtype glioblastomas (including 396 with and 70 patients without **TERT** promotor mutations), we found that **TERT** promotor mutations were neither associated with progression-free survival nor overall survival. This held true in various treatment-based or molecular subgroups. This argues against standardized analysis for **TERT** promotor mutation status for the purpose of prognostic or therapeutic relevance in newly diagnosed **IDH**-wildtype glioblastoma that otherwise meets the histopathological and molecular diagnosis criteria.

The WHO 2021 classification restricts the diagnosis of “glioblastoma WHO grade 4” to *IDH*-wildtype astrocytic gliomas either with (1) classical histopathological hallmarks or (2) qualifying molecular features.^1^ The latter include *EGFR* amplification, +7/−10 genotype, and *TERT* promotor mutation which are all associated with less favorable outcome when observed in combination with *IDH*-wildtype status.^2,3^ The presence of one of these three markers allows the diagnosis of “molecular” glioblastoma even when tumors appear histologically lower grade, and 80% of glioblastomas exhibit *TERT* promotor mutations.^4^ Whether *TERT* promotor mutations are of prognostic value in *IDH*-wildtype glioblastomas which otherwise yet fulfill the diagnostic (histopathological or molecular) criteria for glioblastoma is unclear. Here, we explored such an association based upon a well-annotated glioblastoma cohort from 7 international neuro-oncological centers participating in the RANO *resect* group.

With approval of the ethics committee of the Ludwig-Maximilians-University (Munich, Germany; AZ-21-0996), the RANO *resect* group compiled a retrospective database of newly diagnosed *IDH*-wildtype glioblastomas treated between 2003 and 2022 with a follow-up of ≥3 months.^5^ For the current study, individuals were selected when information on *TERT* promotor mutation status was available for review. Demographics, molecular information, clinical data, and outcome were extracted; and date of progression was determined *per* RANO criteria.

**Figure 1.**
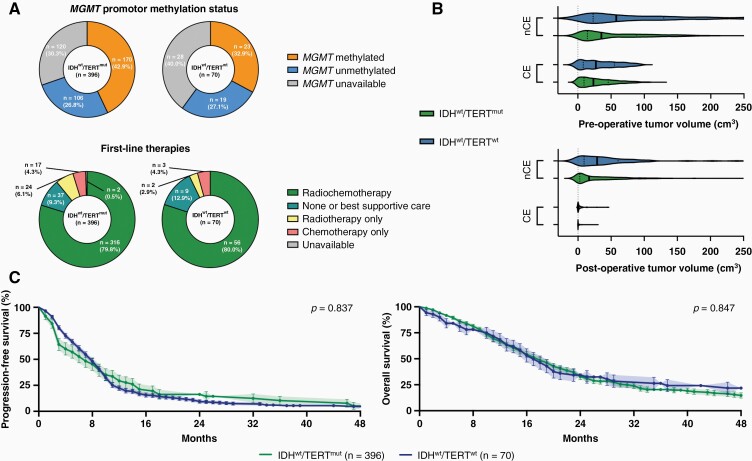
Clinico-molecular markers and outcome in *IDH*-wildtype glioblastoma with or without *TERT* promotor mutations. (A) Distribution of *MGMT* promotor methylation status (upper panel) and first-line therapies following surgery (lower panel) in *IDH*-wildtype glioblastomas with (IDH^wt^/TERT^mut^; *n* = 396) or without *TERT* promotor mutations (IDH^wt^/TERT^wt^; *n* = 70). (B) Pre- (upper panel) and postoperative tumor volumes (lower panel) in cm^3^ among *IDH*-wildtype glioblastomas undergoing microsurgical tumor resection with (IDH^wt^/TERT^mut^; *n* = 358; green) or without *TERT* promotor mutations (IDH^wt^/TERT^wt^; *n* = 63; blue). Volumes are indicated for contrast-enhancing (CE) and noncontrast-enhancing (nCE) tumor tissue. Median ± interquartile range. (C) Kaplan–Meier estimates of progression-free survival (left) and overall survival (right) for *IDH*-wildtype glioblastomas with (green line) or without *TERT* promotor mutations (blue line). Points indicate deceased or censored patients; light shadings indicate SEM.

Among 1008 *IDH*-wildtype glioblastomas WHO grade 4, *TERT* promotor status was available in 466 patients including 396 individuals with (IDH^wt^/TERT^mut^) and 70 patients without *TERT* promotor mutations (IDH^wt^/TERT^wt^). Diagnosis rested upon *IDH*-wildtype combined with histopathological findings in 372 IDH^wt^/TERT^mut^ (93.9%) and 65 IDH^wt^/TERT^wt^ patients (92.9%); and was established based on the molecular signature (*TERT* promotor mutation for IDH^wt^/TERT^mut^; *EGFR* amplification for IDH^wt^/TERT^wt^) in the absence of classical histological findings in the remaining patients. Three hundred and fifty-eight IDH^wt^/TERT^mut^ (90.4%) and 63 IDH^wt^/TERT^wt^ patients (90%) underwent microsurgical resection, whereas the remaining had biopsy for tissue-based diagnosis. There were no differences in *MGMT* promotor methylation status, first-line therapy, or pre- and postoperative tumor volumes (both for contrast-enhancing and noncontrast-enhancing tumor) between IDH^wt^/TERT^mut^ and IDH^wt^/TERT^wt^ patients (Figure 1A and B). Median progression-free survival was 8 months and overall survival was 18 months at a median follow-up time of 36 months (IDH^wt^/TERT^mut^ vs IDH^wt^/TERT^wt^: 33 vs 52 months; HR: 1.50, CI: 1.0–2.3). When patients were stratified according to *TERT* promotor mutation status, no outcome differences were detected for progression-free survival (IDH^wt^/TERT^mut^ vs IDH^wt^/TERT^wt^: 7 vs 8 months; HR: 1.03, CI: 0.8–1.4) or overall survival (IDH^wt^/TERT^mut^ vs IDH^wt^/TERT^wt^: 18 vs 17 months; HR: 0.97, CI: 0.7–1.3) (Figure 1C). Also, no association between survival and *TERT* promotor mutation status was found in the subgroups of patients with *MGMT* promotor methylation (HR for IDH^wt^/TERT^wt^: 0.99, CI: 0.6–1.8), unmethylated *MGMT* promotor status (HR for IDH^wt^/TERT^wt^: 0.92, CI: 0.5–1.7), first-line radiochemotherapy *per* EORTC 26981/22981 (HR for IDH^wt^/TERT^wt^: 1.00, CI: 0.7–1.4), or classical histopathological findings of glioblastoma (HR for IDH^wt^/TERT^wt^: 1.06, CI: 0.8–1.5).

We did therefore not find evidence that *TERT* promotor status adds prognostic information in *IDH*-wildtype glioblastomas exhibiting classical histopathological hallmarks (or other mutations) sufficient for glioblastoma diagnosis. This is in line with previous reports on *IDH*-wildtype glioblastomas,^4,6,7^ although these studies have either not controlled for clinical and molecular confounders^4,6^ or were substantially limited in sample size.^4,7^ Notably, IDH^wt^/TERT^wt^ glioblastomas may identify a subset with a distinct (epi-)genetic and molecular profile compared to IDH^wt^/TERT^mut^ tumors and may benefit from different, personalized treatment strategies.^2,4,6^ These biological findings, however, to date do not result in different clinical outcomes. Thus, up to now our retrospective data argue against standardized analysis for *TERT* promotor mutation status for the purpose of prognostic or therapeutic relevance in newly diagnosed *IDH*-wildtype glioblastoma that otherwise meets the histopathological and molecular diagnosis criteria. This might change in the future whenever *TERT*-directed therapies emerge.
